# Asymmetric-Based Residual Shrinkage Encoder Bearing Health Index Construction and Remaining Life Prediction

**DOI:** 10.3390/s24206510

**Published:** 2024-10-10

**Authors:** Baobao Zhang, Jianjie Zhang, Peibo Yu, Jianhui Cao, Yihang Peng

**Affiliations:** 1 College of Software, Xinjiang University, Urumqi 830091, China; 107552204841@stu.xju.edu.cn; 2 College of Mechanical Engineering, Xinjiang University, Urumqi 830017, China; 107552204321@stu.xju.edu.cn (P.Y.); 107552204151@stu.xju.edu.cn (J.C.); 107552304306@stu.xju.edu.cn (Y.P.)

**Keywords:** bearings, RUL prediction, health indicators, asymmetric autoencoder, deep learning

## Abstract

Predicting the remaining useful life (RUL) of bearings is crucial for maintaining the reliability and availability of mechanical systems. Constructing health indicators (HIs) is a fundamental step in the methodology for predicting the RUL of rolling bearings. Traditional HI construction often involves determining the degradation stage of the bearing by extracting time–frequency domain features from raw data using a priori knowledge and setting artificial thresholds; this approach does not fully utilize the vibration information in the bearing data. In order to address the above problems, this paper proposes an Asymmetric Residual Shrinkage Convolutional Autoencoder (ARSCAE) model. The asymmetric structure of the ARSCAE model is characterized by the soft thresholding of signal features in the encoder part to achieve noise reduction. The decoder part consists of convolutional and pooling layers for data reconstruction. This model can directly construct HIs from the original vibration signals collected, and comparisons with other models show that it constructs better HIs from the original vibration signals. Finally, experiments on the FEMTO dataset show that the results indicate that the HIS constructed by the ARSCAE model has better lifetime prediction capability compared to other methods.

## 1. Introduction

The normal operation of bearings is crucial for ensuring the health and safety of rotating machinery [1]. Studies have shown that factors such as rotational speed, external loads, and surface roughness can affect the normal operation of bearings and, consequently, their fatigue life. Therefore, testing the residual useful life (RUL) of bearings is essential to ensure the safe and stable operation of rotating machinery [2]. Existing RUL prediction methods can be categorized into four groups: physical model-based methods, statistical model-based methods, data-driven methods, and hybrid methods [3]. Physical model-based prediction methods require mathematical modeling to describe the degradation mechanism of bearings; however, this approach is challenging for complex mechanical systems. Statistical modeling approaches rely on empirical knowledge to create statistical models, but they depend on existing observational data and can be affected by inconsistent data distribution [4]. Hybrid methods combine multiple approaches to leverage their respective advantages and improve prediction accuracy, but they still require careful consideration of the applicability of different methods and data characteristics. Data-driven methods, on the other hand, do not require accurate physical models or expert knowledge and have powerful data processing capabilities [5]. With the development of the Internet and big data, deep learning has emerged as an important and effective method among data-driven approaches. Consequently, more and more researchers are applying deep learning in RUL prediction modeling.

Zhang [6] guided the LSTM model by constructing a set of nonlinear HI functions and compressing or stretching the time series to dynamically fuse past feature information through a time window, reducing the adverse effect of long-term memory on RUL prediction; the trained model was then used for RUL prediction. Wang [7] proposed a bearing remaining service life prediction method based on a convolutional attention mechanism and temporal convolutional network (TCN), which adaptively assigns weights in TCN residual blocks to make the prediction network focus more on degraded feature information. The effectiveness of this method was verified using the PHM2012 dataset. Cheng [8] automatically learned features in the bearing data through a fast search discovery density peak clustering method and constructed an RUL prediction model using a parallel bi-directional LSTM and bi-directional gated recurrent unit channel to achieve accurate RUL prediction. Cai [9] proposed a rolling bearing RUL prediction network based on a deep BiLSTM model and a degradation detection strategy. First, time domain, frequency domain, and time–frequency domain features were fully extracted from the bearing signals. The optimal features were selected by constructing a weighted composite index, and the model was optimized for RUL prediction using the Dropout technique and segmented learning rate.

In predicting the RUL of machinery, constructing a health indicator (HI) is a crucial step. HI is used to evaluate the current health of the bearing and its possible future degradation trend [10]. The importance of HI lies in its ability to help predict the RUL of the bearing, which is essential to avoid sudden stoppages of rotating machinery [11]. In previous HI construction methods, the health indicator (HI) of the bearing is usually obtained through signal processing. For example, the root mean square (rms) of the bearing is often used as the HI to predict the remaining life of the bearing [12]. Li [13] used a method based on a chaotic mapping system and low-pass filters (LPFs), extracting features using Euclidean eigenvalues (EFVs) to construct a useful health indicator. Wang [14] extracted 13 time domain features, such as rms, from the original signal and captured the degradation features by calculating these features. Liu [15] selected 11 statistical features, such as kurtosis, to be input into a support vector regression network, which was then used to compare the RUL prediction accuracies of the bearings. The core of the methods mentioned above is to extract useful information from vibration signals. However, these methods do not account for the nonstationary nature of vibration information. In recent years, new approaches have emerged, such as data-driven health indicator construction methods [16], distance metric learning (DML)-based health indicator construction methods [17], and new methods incorporating machine learning techniques aimed at improving the accuracy and reliability of health indicators [18]. Among them, the data-driven approach is more advantageous in constructing bearing health metrics by modeling the results without the need to go through human calculations or a priori knowledge. Guo [19] proposed combining six relevant similar features with eight classical time–frequency features to form an original feature set, from which the most sensitive features are selected; these selected features are then fed into a recurrent neural network to construct the RNN-HI. Another study [20] used self-organizing mapping (SOM) to fuse the extracted features to construct the HI of rolling bearings. In the study of Ding [21], the extracted signal is first converted to a time–frequency image by time domain analysis. These images are then introduced into the constructed model for training and HI construction. Finally, the RUL failure point of the bearings is determined by calculating the composite index to predict the RUL. Autoencoder (AE) has the ability to learn effective data encoding through unsupervised learning. Therefore, AE can be used to learn low-dimensional information that contains the most significant data, making them applicable for constructing health indicators (HIs) for mechanical devices, gears, and bearings [22]. Lin [23] proposed integrated stacked self-encoders to construct bearing HIs. Four different self-encoders were utilized to extract features by selecting the vibration spectrum, and the extracted features were then used to train the model and extract HIs. Chen [24] proposed a quadratic function-based method for HI construction using self-encoders. The results showed that the constructed HI could better reflect the degradation of the bearing compared to traditional degradation functions. Ye [25] proposed a multi-scale convolutional self-encoder method, which fully utilizes both global and local information of the vibration data. This method extracts the HI through the parallel composition of three convolutional self-encoders with different convolutional kernel sizes and finally uses an LSTM neural network to perform RUL prediction, verifying the superiority of the extracted HI. Several problems still exist in the above work. However, there are still some shortcomings in the above working methods. When constructing the HI, few methods are used to obtain the HI from the original vibration signals, but the time–frequency domain information of the vibration signals is first processed, and the extracted features are then used as model inputs. In addition, in the traditional convolutional self-encoder design, although it is possible to directly input the original vibration signal as features, the noise in the original vibration signal will affect the model. The noise in the original vibration signal will affect the feature extraction ability, and this problem will make the model not necessarily effective in extracting the HI under the noise interference. To solve these problems, the contributions of this paper are as follows:

1. We propose the use of the ARSCAE model to construct HI. Compared with the traditional HI construction method, this method does not need a manual way for feature extraction, and realizes the automatic extraction of HI directly from the original vibration signal.
2. For the encoder structure by asymmetric structural design, a one-dimensional residual shrinkage convolutional self-encoder based on convolutional encoder is designed to introduce soft thresholding to minimize the noise interference, while the decoder structure remains unchanged.
3. The superiority of the extracted HI is demonstrated by using the proposed HI construction method in several bearings for experiments and also for RUL prediction of the extracted HI.

The rest of the paper is organized as follows. Section 2 describes the methodology and basic theory required for modeling asymmetric residual shrinkage convolutional encoders. Section 3 introduces the model of this paper and describes the algorithmic procedure. Section 4 demonstrates the validity of the proposed methodology by conducting an experimental study with the dataset FEMTO. Section 5 performs RUL prediction with the HI extracted from the model. Finally, Section 6 draws conclusions and summarizes.

## 2. Theoretical Background

### 2.1. Autoencoder

**Figure 1:**
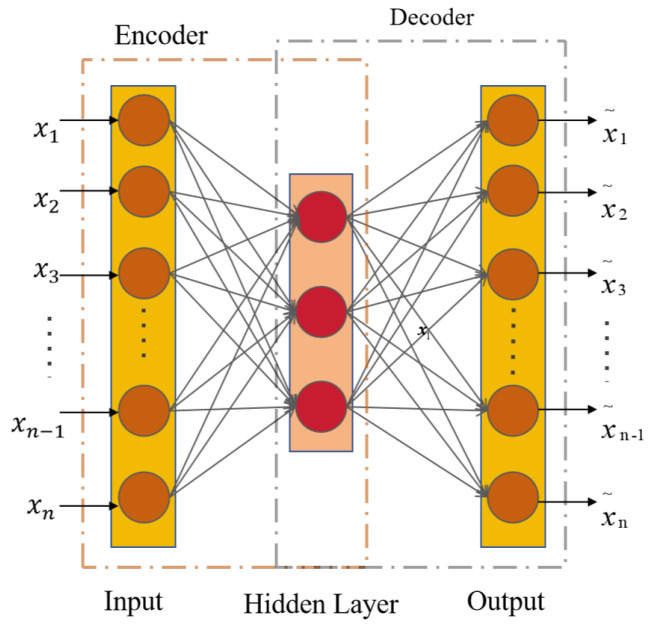
The architecture of AE.

Autoencoder (AE) [26] is a neural network used for the unsupervised learning of compressed data representations. The system comprises two main components: an encoder and a decoder. The encoder compresses the data to extract low-dimensional features that retain the original information. The decoder then reconstructs these low-dimensional features back into the original data space. During training, the goal of AE is to computationally reduce input data and reconstruction data errors to ensure that the model captures and retains the most critical information in the input data. The network structure of AE is shown in Figure 1. In the computation of the AE, let the encoder’s input be denoted as $x=\left[x_{1},x_{2},x_{3},\ldots ,x_{i}\right]$, where i represents the length of the input data. The encoder’s output can then be expressed as follows:

(1) $$E=F_{e}\left(W_{e}x+b_{e}\right)$$

here, $W_{e}$ denotes the encoder’s weights, $b_{e}$ signifies the encoder’s bias, and $F_{e}$ stands for the encoder’s activation function. The output of the encoder is represented by *E*. The decoder output result can be expressed as

(2) $$D=F_{d}\left(W_{d}x+b_{d}\right)$$

where $W_{d}$ and $b_{d}$ represent the weights and biases, respectively, while $F_{d}$ indicates the activation function of the decoder. During training, the AE optimizes its model parameters by minimizing the differences between the input data and the reconstructed data. The reconstruction error *L* can be expressed as

(3) $$L_{Ae}=\frac{1}{l}\sum_{i=1}^{l}F\left(D_{i},E_{i}\right)$$

where *F* denotes the loss function used to compute the reconstructed data and the input data.

### 2.2. Deep Residual Shrinkage Network

**Figure 2:**
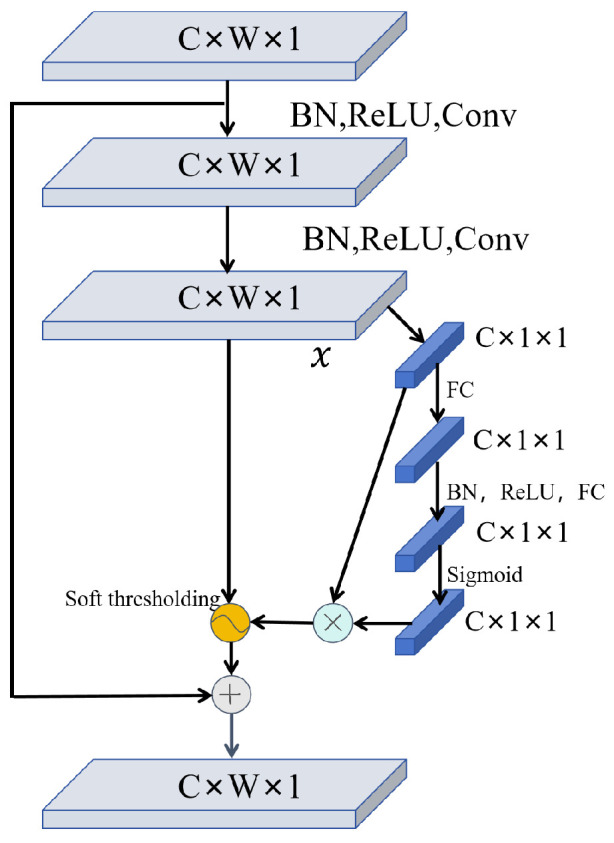
Residual shrinkage network.

The residual Shrinkage network model is a further development of the network structure based on the CNN, which introduces residual connectivity and Shrinkage modules to enhance the depth and performance of the network, and combines the concepts of residual learning and network contraction [27]. As illustrated in Figure 2, the deep residual contraction network incorporates an attention mechanism and a soft threshold function, building upon the residual contraction network. A key innovation in deep residual shrinkage networks is automatic thresholding. The soft threshold function is a nonlinear function that adjusts the eigenvalues within the threshold interval to zero and shrinks other eigenvalues. The principle is to scale the values to a certain range, thereby achieving noise reduction. This method retains most of the information of the signal while removing the noise. During gradient operations, the derivative of the soft threshold function being 1 or 0 effectively mitigates gradient vanishing and explosion issues. By integrating the residual shrinkage module, the network can capture long-term dependencies in sequence data during feature extraction, enhancing both representation and generalization capabilities.

## 3. Methods

In this section, we will explain the constructed ARSCAE model architecture and describe the HI construction process.

### 3.1. Construction of the ARSCAE Model

**Figure 3:**
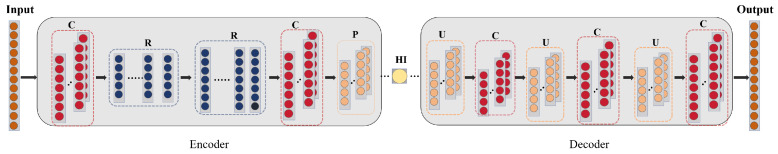
The structure of DCAE.

Convolutional encoder is formed by introducing multiple convolutional layers on top of the self encoder. This approach allows the convolutional encoder to leverage the benefits of the self-encoder in downsizing and feature extraction, while also enhancing the model’s generalization ability and efficiency through the utilization of CNNs’ local connectivity and weight sharing properties [28]. Based on the convolutional encoder, to capture more complex features and enable adaptive feature extraction, this paper introduces an asymmetric structural encoder [23,29] to learn distinct encoding and decoding weights. While traditional self-encoders exhibit a symmetric structure where the encoder part is used to extract features while the decoder part is used to reduce the feature data, asymmetric encoders focus on the encoder part being used to extract the features, and the decoder structure adopts an alternative structure to accomplish the reconstruction task. This correct asymmetric structure ensures that the encoder can reduce computational and time overheads and improve the accuracy and efficiency of the model. For the input raw signal, the encoder part has to extract feature information while considering the influence of noise; therefore, we have designed a more intricate structure for the encoder part. On the other hand, the decoder’s role is to restore the data, so its structure has been streamlined to minimize memory usage and accelerate training overall. In the actual design of the ARSCAE model in this study, the encoder section’s convolutional network module initially conducts feature extraction using a convolutional layer, and subsequently diminishes noise in the feature signals through the integration of a residual shrinkage network. Meanwhile, in the decoder section, we utilize the convolutional network module to achieve data reconstruction. The ARSCAE structure designed in this study is illustrated in Figure 3.

The encoder part includes a convolutional layer (C), a residual shrinkage module (R) and a pooling layer (P). The decoder part includes the convolutional layer and the upsampling layer (U). In this model, the convolutional operation formula can be expressed as

(4) $$x_{j}^{l}=f\left(\sum_{i\in M_{j}}x_{i}^{l-1}k_{ij}^{l}+b_{j}^{l}\right)$$

given that $M_{j}$ represents the input eigenvector, *l* denotes the *l*-th layer in the network, *k* signifies the convolution kernel, *b* stands for the network bias, $x_{j}^{l}$ is the output of the *l*-th layer, and $x_{i}^{l-1}$ is the input of the preceding layer *l*-1th. In the training process, the convolutional layer first initializes the convolutional kernel and bias terms; the convolutional kernel slides and extracts the features during forward propagation and updates the parameters by calculating the error through back propagation, which is ultimately repeated several times until the network converges. Typically, a pooling layer is used after one or more CNN layers to provide invariance by reducing the resolution of the feature mapping. After performing convolution operation on the input data, the downsampling operation is performed using the maximum pooling function to reduce the size of the data. The maximum pooling function can be expressed as

(5) $$P_{i}^{l+1}\left(j\right)=\max_{(j-1)V+1\leq n\leq jV}\left\{q_{i}^{l}\left(n\right)\right\}$$

and in this context, $q_{i}^{l}\left(n\right)$ denotes the value of the *n*-th neuron within the *l*-th eigenvector of the *i*-th layer, where *n* ranges from n∈[(j−1)V+1,jV]. Here, *V* represents the width of the pooling area, and $P_{i}^{l+1}\left(j\right)$ signifies the corresponding value of the neurons in the (l+1)-th layer. After performing the convolution and maximum pooling operation on the data, the feature dimension is changed to one dimension by the flatten function and the output is obtained by the nonlinear activation function sigmoid. In the decoder section, the extracted HI is subjected to a dimensional unfolding operation using convolution and upsampling operations, and the upsampling formula can be defined as

(6) $$U_{n.m}^{i}=\mathrm{unsampling}\left(I_{n,m}^{i}\right)$$

where $I_{n,m}^{i}$ denotes the input of point *I* at the *n*-th block of data in layer *m*. $U_{n,m}^{i}$ denotes the upsampling layer output of point *i* at the *n*-th block of data in layer *m*. For the input data X$=\left[x_{1},x_{2},x_{3},\ldots ,x_{n}\right]$ is reconstructed through the model to obtain $X^{\prime }=\left[x_{1}^{\prime },x_{2}^{\prime },x_{3}^{\prime },\ldots ,x_{n}^{\prime }\right]$, then the loss function of ARSCAE can be defined as

(7) $$L=\frac{1}{n}\sum_{j=1}^{n}∥X_{j}-X_{j}^{\prime }∥$$

### 3.2. Health Trend Setting

**Figure 4:**
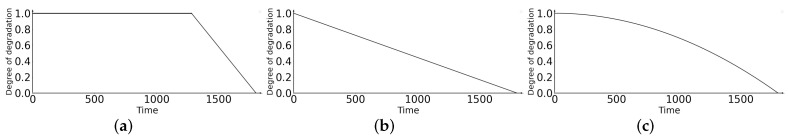
Three primary methods for constructing degradation labels. (**a**) segmented function. (**b**) segmented function. (**c**) quadratic function.

After obtaining the raw vibration signals, the degradation trend in the bearing condition sink is determined by analyzing the vibration signals. Studies have shown that bearings tend to degrade differently in different environments [30], so it is necessary to ensure that the selected degradation trend indexes are consistent with the degradation condition of bearings in multiple environments. Two primary traditional methods exist for constructing degradation indicators: the linear function and the segmented function. The equation for constructing the degradation trend label, as shown in Figure 4a, is formulated as follows:

(8) $$R_{i}=1-\frac{t_{i}}{T}$$

where *T* is the total running time of the bearing, $t_{i}$ denotes the current operating time of the bearing, and $R_{i}$ indicates the current remaining operating life of the bearing. The equation for constructing the degradation trend label, as shown in Figure 4b, is formulated as follows:

(9) $$R_{i}=\left\{\begin{array}{ccc} 1 & \;\mathrm{if}\; & t_{i}\leq t_{s}\\ x & \;\mathrm{if}\; & t_{i}>t_{s} \end{array}\right.$$

where $t_{s}$ represents the degradation threshold of bearing life when $t_{i}$ is less than or equal to $t_{s}$. This indicates that the bearing has not yet entered the degradation stage, so the degradation label is set to 1, signifying that the bearing is in a normal state. Once the specified threshold ts is surpassed, the bearing begins to exhibit a linear degradation trend.

Both of the traditional methods described above characterize the degradation of a bearing by means of a linear function. However, the linear indicator setting can only exist in the ideal situation; in practice, the operating state of the bearing does not conform to the linear degradation but presents a kind of nonlinear degradation, so the traditional indicator setting has certain limitations. Chen [24] introduced a method for constructing a bearing degradation trend indicator using a quadratic function. The specific formula, illustrated in Figure 4c, is as follows:

(10) $$R_{i}=1-\frac{t_{i}^{2}}{T^{2}}$$

This degradation trend construction method eliminates the need for artificially set thresholds while ensuring that lifetime degradation has a nonlinear character. Hence, this study employs the quadratic function approach to develop the degradation index for bearings.

### 3.3. Model Flow Procedures

**Figure 5:**
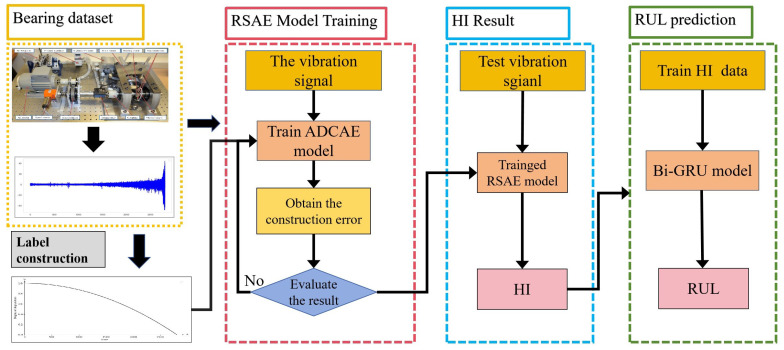
HI extraction and RUL prediction process.

This subsection describes the process structure of HI extraction and RUL prediction. As shown in Figure 5 the specific method steps are as follows.

1. Input raw vibration data $Y=\left[y_{1},y_{2},y_{3},\ldots ,y_{n}\right]$ denote the original vibration signal dataset, where $y_{i}=\left[y_{i1},y_{i2},y_{i3},\ldots ,y_{il}\right]$. In this equation, *n* represents the length of the vibration samples, while *l* indicates the number of each sample.
2. Using the quadratic indicator formula proposed in the previous section, the trend indicator is calculated for the processed vibration signal to obtain $H=\left[h_{1},h_{2},h_{3},\ldots ,h_{n}\right]$.
3. Input the training set *Y* and the degradation trend *H* into the ARSCAE model to participate in training. During training, the model outputs two results, HI and *D*. HI, as the encoder’s output, is represented as the health indicator, and $\bar{Y}$ is represented as the decoder’s reconstructed output of the health indicator. Finally, the two results are used to compute the error by the composite loss function. The formula is as follows, where Loss is the loss value, *w* is the scale factor, *Y* denotes the input data, $\bar{Y}$ denotes the data output by the decoder in the model, HI denotes the health indicator index data output, and *H* denotes the degradation index data constructed using the quadratic function. Throughout the training process, the weights and parameters in the ARSCAE network are updated by minimizing the loss function from the training dataset:
  (11) $$\;\mathrm{Loss}\;=\frac{1}{N}\sum_{i=1}^{N}{∥HI_{i}-H_{i}∥}^{2}+w\frac{1}{N}\sum_{i=1}^{N}{∥Y_{i}-\bar{Y_{i}}∥}^{2}$$
4. The original vibration signal of the input test set is T$=\left[t_{1},t_{2},t_{3},\ldots ,t_{i}]\right.$ where $t_{j}=\left[t_{i1},t_{i2},t_{i3},\ldots ,t_{im}\right]$. Subsequently, the test set *T* is input into the trained ARSCA model, producing the health indicator $\mathrm{HI}=\left[HI_{1},HI_{2},\ldots ,HI_{m}\right]$ for the test set.
5. After obtaining the HI of the bearing, assume that the extracted HI sequence is $H=\left[h_{1},h_{2},h_{3},\cdots ,h_{N}\right]$, let the last *k* points of H be unknown, then the HI sequence is known to be $H=\left[h_{1},h_{2},h_{3},\cdots ,h_{N-K}\right]$. Construct the training matrix *V*, which can be expressed as
  (12) $$V=\left[\begin{array}{cccc} h_{1} & h_{2} & \cdots & h_{N-K-M}\\ h_{2} & h_{3} & \cdots & h_{N-K-M+1}\\ \vdots & \vdots & \ddots & \vdots \\ h_{M+1} & h_{M+2} & \cdots & h_{N-K} \end{array}\right]=\left[\begin{array}{c} v_{1}\\ v_{2}\\ \vdots \\ v_{M+1} \end{array}\right]$$
  In predicting RUL, the first *v* vectors of the training matrix *M* are used as inputs to the BI-GRU neural network, and the last vector $V_{m+1}$ is used as the output. After training the model, the last *M* vectors of matrix *v* are used as inputs for the trained BI-GRU neural network. The prediction result is then obtained, and the matrix is updated. If the prediction result exceeds the set threshold, the prediction stops, and the RUL value is determined.

## 4. Experiments

In order to validate the performance of the method in this paper, we conduct experiments using the proposed method on the bearing dataset of PRONOSTIA to verify the effectiveness of the method.

### 4.1. Dataset

**Figure 6:**
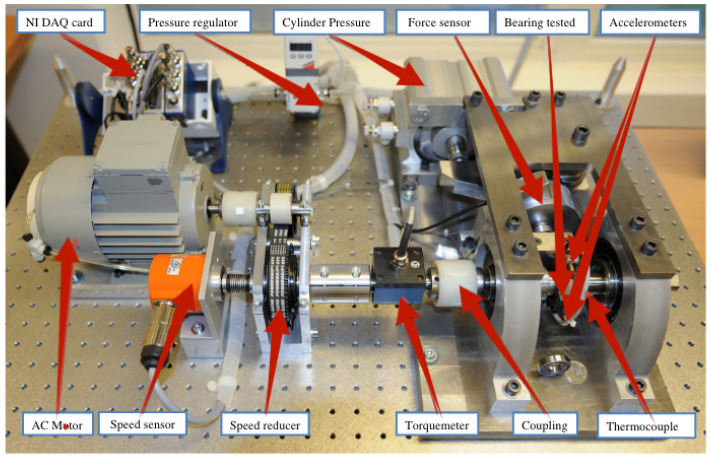
PRONOSTIA experimental platform.

This study uses the IEEE 2012 Prognostics and Health Management Data Challenge dataset to validate our proposed method. As shown in Figure 6, horizontal vibration and vertical vibration signals are provided with a sampling frequency of 25.6 kHz, and the sampling process is to record 2560 samples by sampling 1 s of data every 10 s. Vibration signal datasets are obtained from horizontal and vertical signal transducers for 17 full life cycles of bearings under three operating conditions. This article explains the dataset in more detail [31].

In this paper, the detailed experimental information and datasets are listed in Table 1. Bearing1_1, Bearing1_3, Bearing 2_5 and Bearing3_3, which are operated to failure under each condition, are selected to construct the test dataset, and the rest of them are constructed as the training dataset.

### 4.2. Evaluation Indicators

To evaluate the model’s effectiveness in constructing HI, appropriate evaluation metrics are required. Therefore, three evaluation metrics are introduced [23]: monotonicity, correlation, and robustness. Additionally, we use polynomial fitting to decompose HI into mean trend and random components. The decomposition equation is as follows:

(13) $$H\left(t_{n}\right)=H_{T}\left(t_{n}\right)+H_{R}\left(t_{n}\right)$$

In this equation, $H\left(t_{n}\right)$ represents the value of HI at time $t_{n}$, $H_{T}\left(t_{n}\right)$ indicates its average trend, and $H_{R}\left(t_{n}\right)$ signifies the random component. The three HI evaluation metrics, correlation (Corr), monotonicity (Mon), and robustness (Rob), are denoted:

(14) $$I_{\mathrm{Mon}\;}\left(HI\left(t_{k}\right)\right)=\left|\frac{N_{\mathrm{positive}\;}-N_{\mathrm{negative}\;}}{K-1}\right|$$

(15) $$I_{\mathrm{Corr}\;}\left(H\right)=\frac{\left|\sum_{k=1}^{k}\left(HI\left(t_{k}\right)-\bar{HI}\right)\left(T\left(t_{k}\right)-\bar{T}\right)\right|}{\sqrt{\sum_{k=1}^{k}{\left(HI\left(t_{k}\right)-\bar{HI}\right)}^{2}\sum_{k=1}^{k}{\left(T\left(t_{k}\right)-\bar{T}\right)}^{2}}}$$

(16) $$\mathrm{Rob}\left(H\right)=\frac{1}{K}\sum_{1}^{k}\exp\left(-\left|\frac{H_{R}\left(t_{n}\right)}{H\left(t_{n}\right)}\right|\right)$$

To evaluate the overall capacity of the HI, a composite index (CI) incorporating all three indices is defined as follows:

(17) $$\mathrm{CI}=\frac{1}{3}\left(\mathrm{Corr}+\mathrm{Mon}+\mathrm{Rob}\right)$$

### 4.3. Results and Comparison of Experiments

**Figure 7:**
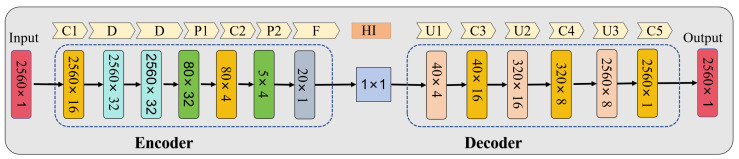
The specific structure of ARSCAE model.

**Figure 8:**
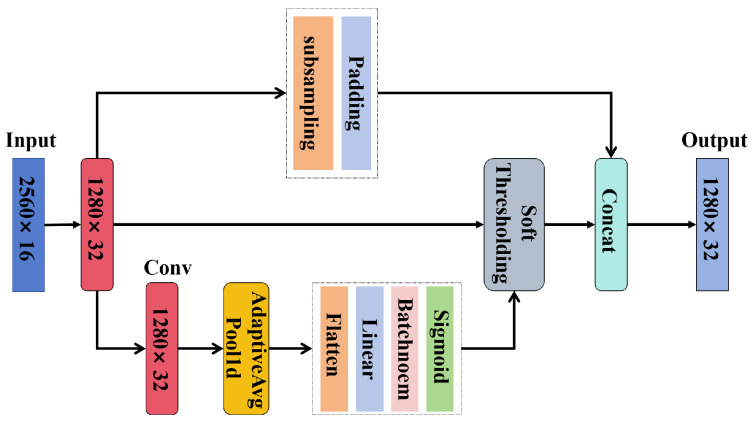
Flow of HI extraction and RUL prediction.

The detailed architecture of the ARSCAE model presented in this paper is illustrated in Figure 7. The encoder component comprises two convolutional layers, two residual shrinkage modules, and a fully connected module. In the encoder section, the two convolutional layers utilize 5 and 11 convolution kernels, respectively. The two residual shrinkage blocks employ convolution kernels of size 3 and a pooling layer size of 16. The detailed structure of the residual shrinkage module is shown in Figure 8.

As shown in Figure 7, in the decoder section, the numbers of convolutional kernels in the convolutional layer are 11, 7, and 3, respectively, and the upsampling kernel size is 8. The specific model parameters are given in Table 2. In the parameter selection of the number of convolutional kernels, we compare the different numbers of convolutional kernels with the specific experimental parameters shown in Table 3. The table contains the number of convolution kernels under three different choices. And the checkmarks and crosses in the table represent whether we got the best results in the experiment. By choosing different numbers of convolution kernels through the model, the average CI result values of 0.87, 0.83 and 0.80 were obtained for the three working conditions, and the most appropriate model parameters were selected through the CI metrics of the different results.

After constructing the model, the parameters must be optimized for various operating conditions, including the model’s learning rate and the scale factor in the composite loss function. In this study, the learning rate is set to 0.0001, and the number of training epochs is fixed at 150. The value of the scaling factor *w* in the loss function lies between 0 and 1. Therefore, five values of 0.2, 0.4, 0.6, 0.8, and 1 are selected for experimentation and comparison. Also, 10 experimental comparisons are performed for each selected *w*. Finally, the best value of *w* under each datum is selected based on the results of the test set and the results are compared by box plots.

**Figure 9:**
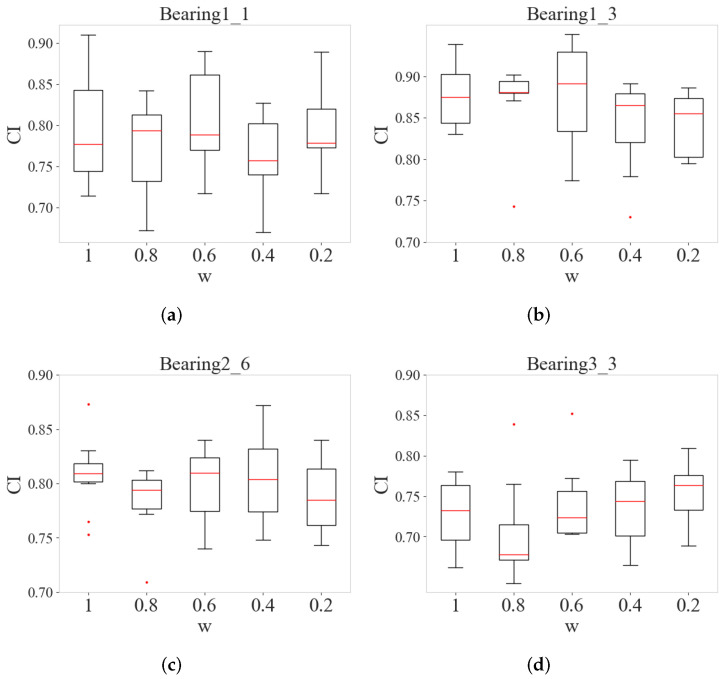
Composite indexes obtained by multiple learning rates. (**a**) bearing1_1 CI values. (**b**) bearing1_3 CI values. (**c**) bearing2_6 CI values. (**d**) bearing3_3 CI values.

**Figure 10:**
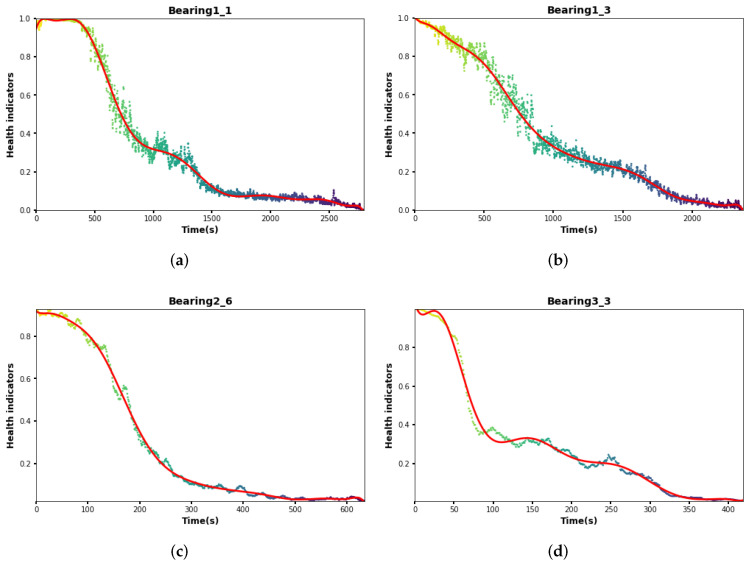
The HIs of test bearings. (**a**) bearing1_1 HI. (**b**) bearing1_3 HI. (**c**) bearing2_6 HI. (**d**) bearing3_3 HI.The scattered dots represent HI values and red lines represent fitting results.

**Figure 11:**
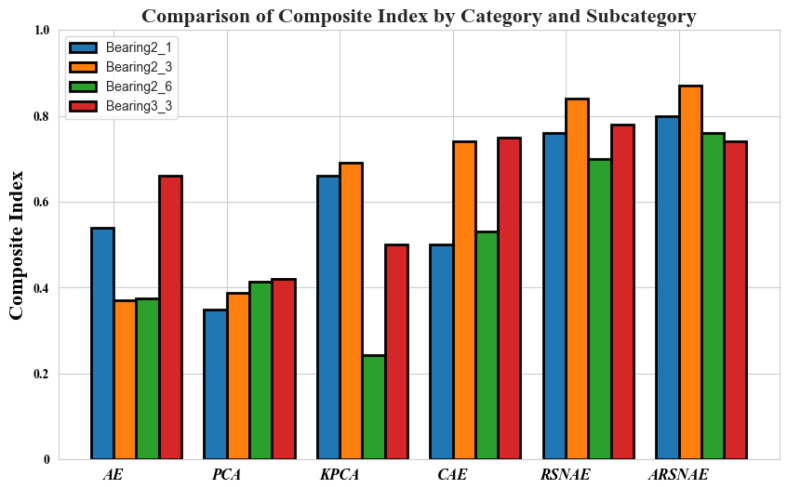
The composite index of different methods.

As shown in Figure 9, the optimal scaling factors under each dataset are 0.8, 0.6, and 0.2, respectively. After selecting the optimal scaling factor for each condition, the HIS results under each test set are obtained as shown in Figure 10. These curves contain HIS values under the test sets Bearing1_1, Bearing1_3, Bearing2_6, and Bearing3_3 with 2799, 2371, 632, and 420 HI points, respectively. From all the HI curves, it is easy to see that with the passage of time, the HI value gradually decreases, which can indicate that the bearing life gradually decreases with the passage of time. At the same time, the resulting HI curves are subjected to the CI index calculation and compared with the CI results derived from other models. In the experiments, five HI construction methods, AE, KPCA, PCA, CAE, and ARSCAE models with symmetric structure (RSCAE), are used for HI construction, and the comparison is evaluated by the Ci index introduced above. The RSCAE model is to maintain that the encoder and decoder have symmetry, with the decoder part corresponding to the encoder part. As shown in Figure 11, the CI values of the ARSCAE model are higher than those of the AE, KPCA, PCA, and CAE models. This is because the introduction of the quadratic trend indicator calculated through the composite loss function ensures that the constructed HIS has a degradation trend, which is more advantageous than the traditional unsupervised methods. Meanwhile, the residual module in the ARSCAE model serves to denoise the features in the encoder part, while the decoder part performs feature reduction through the inverse convolutional layer, which eliminates the effect of the residual module on the features in the decoder part. In order to validate the superiority of the designed asymmetric structure, it is necessary to experimentally compare the asymmetric model structures; thus, further exploration of the ARSCAE and RSCAE models is needed to validate the superiority of the structural choices.

Meanwhile, the RSCAE model needs to be described in detail in the comparison. The RSCAE model continues the symmetry of the AE model. The encoder part of the RSCAE model is the same as that of the ARSCAE model, while the decoder part remains symmetric with the encoder part. In the RSCAE model, the residual shrinkage module is also referenced in the decoder section, so it is necessary to explore whether the residual shrinkage module plays a role in the decoder section. For the RSCAE model, the same HI construction method using quadratic trend labeling is used for HI construction while keeping the same parameters as the ARSCAE model. As shown in Figure 11, in Beaing3_3, the CI metrics of the symmetric structure are slightly higher in effect than the results derived from the asymmetric structure, but in general, the average CI metrics of the models with the asymmetric structure are higher than the average CI metrics derived from the symmetric model. This can also indicate that the ARSCAR model generalizes better than the RSCAR model. In order to verify the superiority of the asymmetric structure even further, we need to experimentally compare the test speeds of the ARSCAE model and the RSCAE model. The experimental results are shown in Table 4.

## 5. RUL Prediction

After the HI vector is obtained from the real bearing dataset, a suitable prediction model needs to be selected for RUL prediction. HI as a time series can be predicted by using a temporal network, which is well solved by a variant based on the RNN model. The GRU model, as a variant of RNN, solves the gradient vanishing problem by introducing a gating mechanism, while the BI-GRU model introduces a bi-directional structure on top of the GRU model to better capture the bi-directional dependency of sequence data. Therefore, this paper uses the BI-GRU model to accomplish RUL prediction. After obtaining the HI vector, for Bearing1_1, we select the previous 2699 points as the training set and the remaining 100 points as the prediction value, and at the same time set the failure threshold of the bearing to 0; when the prediction value is equal to 0, it represents that the life of the bearing is going to decline. Similarly for Bearing1_3, we select the first 2271 points as the training set and predict the last 100 points. The specific prediction model parameters are given in Table 5. The selection of the predicted remaining life values indicates the applicability of the constructed HI results. In the selection of the life prediction values, the experiment determines the most appropriate remaining prediction values by comparing the MAE values of the predicted remaining life of 50, 100, and 150 points, and the results obtained are shown in Table 6. Therefore, in this paper, we choose to predict the 100 remaining life values. In order to verify the superiority of HI constructed by the ARSCAE model, the HI results based on the quadratic function CAE model [25] and RSCAE are compared. In addition, the RUL results predicted by the model are validated by calculating three indicators: RMSE, MAE, and NRMSE.

**Figure 12:**
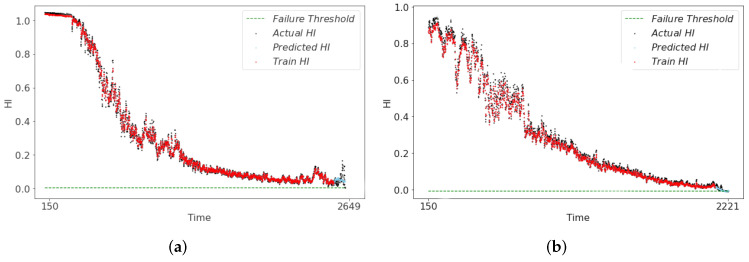
RUL prediction results of RSCAE-HI. (**a**) bearing1_1 RUL result. (**b**) bearing1_3 RUL result.

**Figure 13:**
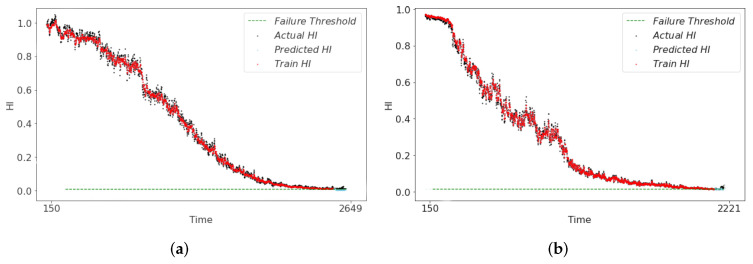
RUL prediction results of CAE-HI. (**a**) bearing1_1 RUL result. (**b**) bearing1_3 RUL result.

**Figure 14:**
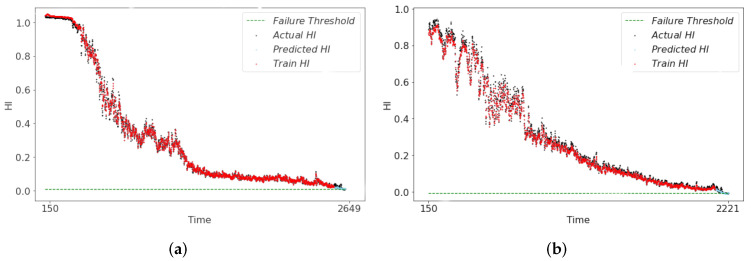
RUL prediction results of ARSCAE-HI. (**a**) bearing1_1 RUL result. (**b**) bearing1_3 RUL result.

The RUL predictions derived from the health indicators (HI) of the three models are illustrated in Figure 12, Figure 13 and Figure 14. In Figure 12, it can be seen that the RUL predictions of the symmetry model structure at the end of the degradation of both Bearing1_1 and Bearing1_3 show an upward trend deviating from the actual degradation thresholds, which is not in line with the actual bearing operating conditions. In the CAE-predicted RUL shown in Figure 13, although the overall result trendiness is relatively smooth, at the end of the degradation period, the RUL prediction results in the early occurrence of the threshold value of 0. This result will lead to the early end of the bearing life. This discrepancy results in the predicted RUL being underestimated compared to the actual RUL value. The HI constructed by the asymmetric structure model has a smoother trend in the life degradation stage as shown in Figure 14. The prediction results effectively capture the actual degradation trend of the bearing and ensures the authenticity of RUL when RUL is predicted. Also, to further illustrate the superiority of the asymmetric structure for building our HI, we use three metrics, MAE, Normalized Root Mean Square Error (NRMSE), and RMSE, for the comparative calculations, and the final results of the evaluation are shown in Table 7. The results show that the asymmetric structure is optimal in the calculation of MAE, NRMSE, and RMSE, verifying the superiority of HI.

## 6. Conclusions

In this paper, we propose a new HI extraction method, namely, the ARSCAE model. This model does not require signal analysis of the raw vibration data and only requires the input of the original signal. In the feature extraction encoder module, the model utilizes a convolutional layer to extract global and local information while introducing soft thresholding for noise reduction in the extracted signal features. Also in this paper, the model is designed as an asymmetric structure, presenting the decoder and encoder asymmetrically. The data are reconstructed using convolution and pooling modules in the decoder part, which enables the model to adaptively learn the encoding and decoding weights and can better adapt to different types of data to extract more effective features. Finally, the validity of the present model is verified experimentally on the PHM2012 dataset. It outperforms AE, CAE, KPCA, and AE obtained by PCA in terms of HI extraction ability, and also compares the symmetric model structure, and the results show that it is higher than the symmetric structure in terms of the HI extraction ability. Finally, the BiGRU model is utilized for RUL prediction, and the results show that the method proposed in this paper fetches good results in both HI extraction and RUL prediction.

## Figures and Tables

**Table 1:** Training set and test set.

Working Condition	Condition1	Condition2	Condition3
Training dataset	Bearing1_3	Bearing2_1	Bearing3_1
	Bearing1_4	Bearing2_2	Bearing3_2
	Bearing1_5	Bearing2_3	
	Bearing1_6	Bearing2_4	
	Bearing1_7	Bearing2_5	
		Bearing2_7	
Test dataset	Bearing1_1		
	Bearing1_2	Bearing2_6	Bearing3_3

**Table 2:** Parameter setting of the ARSCAE.

Parameters	Value
Batch size	128
Epoch	150
Number of DRSN Block	2
Learning rate lr	Adam (0.0001)
Encoder Kernel size	[5 × 1] [3 × 1] [11 × 1]
Decoder Kernel size	[11 × 1] [7 × 1] [3 × 1]
Pooling size	16
Upsampling size	8

**Table 3:** Choice of number of convolution kernels.

	Number of Convolution Kernels				
	[5 × 1]	[3 × 1]	[11 × 1]	[7 × 1]	Select
	1	1	1	0	✓
Encoder	1	1	0	1	×
	0	1	1	1	×
	0	1	1	1	✓
Decoder	1	1	1	0	×
	1	1	1	0	×

**Table 4:** Speed comparison between ARSCAE and RSCAE models.

Time/s			
	Condition1	Condition2	Condition3
ARSCAE	145.3	117.6	53.2
RSCAE	232.6	171.2	75.8

**Table 5:** Parameter setting of the BI-GRU model.

Parameters	Value
input layer	150
hidden layer	50
output layer	50
Learning rate lr	0.001
Optimizer	Adam

**Table 6:** Model Predicted Life Value Selection.

MAE			
	50	100	150
Bearing1_1	0.021	0.007	0.013
Bearing1_3	0.032	0.006	0.025

**Table 7:** Results of RUL prediction evaluation for HI.

MAE			
	ARSCAE	RSCAE	CAE
Bearing1_1	0.007	0.022	0.012
Bearing1_3	0.006	0.046	0.035
NRMSE			
	ARSCAE	RSCAE	CAE
Bearing1_1	0.288	0.51	0.293
Beainrg1_3	0.221	0.55	0.255
RMSE			
	ARSCAE	RSCAE	CAE
Bearing1_1	0.009	0.032	0.010
Beainrg1_3	0.008	0.061	0.015

## Data Availability

Data are contained within the article.
